# Epidemiology and outcomes of ventilator-associated pneumonia in northern Brazil: an analytical descriptive prospective cohort study

**DOI:** 10.1186/1471-2334-13-119

**Published:** 2013-03-05

**Authors:** Marília M Resende, Sílvio G Monteiro, Bianca Callegari, Patrícia M S Figueiredo, Cinara R A V Monteiro, Valério Monteiro-Neto

**Affiliations:** 1 Faculdade Estácio Seama, Macapá, Amapá, Brazil; 2 Laboratório de Microbiologia, Universidade CEUMA, São Luís, Maranhão, Brazil; 3 Departamento de Ciências Biológicas, Universidade Federal do Maranhão, São Luís, Maranhão, Brazil; 4 Instituto de Ciências da Saúde, Universidade Federal do Pará, Belém, Pará, Brazil; 5 Departamento de Patologia, Universidade Federal do Maranhão, São Luís, Maranhão, Brazil; 6 Pró-Reitoria de Pós-Graduação, Pesquisa e Extensão, Universidade CEUMA, Rua Josué Montello No.1, CEP: 65.075-120, São Luís, MA, Brazil

**Keywords:** Ventilator-associated pneumonia, Invasive mechanical ventilation, Bacterial multiresistance, Epidemiology

## Abstract

**Background:**

Ventilator-associated pneumonia (VAP) is considered the most common nosocomial infection in the intensive care unit (ICU), but its features are not fully known in many hospitals in Brazil. We identified clinical and epidemiological aspects associated with VAP in an intensive care unit (ICU) in a general public hospital in northern Brazil and performed an analytical descriptive prospective cohort study.

**Methods:**

We analyzed data from thirty-three patients who developed VAP while in the ICU. Clinical and epidemiological data of patients were obtained and tracheal secretions were submitted to culture. Microbial isolates were identified and evaluated for resistance against antimicrobial agents by using the automated Vitek 2 system.

**Results:**

The frequency of VAP was 26.2% in patients submitted to invasive mechanical ventilation for at least 48 hours, and death occurred in 78.8% of cases. Only the presence of comorbidity showed a significant association (*P* = 0.029) with death. The most commonly found bacteria were *Pseudomonas aeruginosa, Acinetobacter* spp*.,* and Enterobacteriaceae*.* We also found a frequency of 54.5% of multiresistant bacteria associated with VAP, and previous antibiotic therapy was used in 97% of patients.

**Conclusions:**

VAP in our ICU presented with a high frequency and was mainly caused by multiresistant bacteria. Implementation of rational protocols for the use of antibacterial agents and rapid delivery of culture and susceptibility test results are essential. This may help decrease VAP-related mortality rates by multiresistant bacteria in the ICU.

## Background

Ventilator-associated pneumonia (VAP) is a pulmonary infection that appears after 48 hours of endotracheal intubation and when invasive mechanical ventilation (IMV) is initially used [1]. VAP is considered the most common nosocomial infection in the intensive care unit (ICU), with an incidence that varies between 9% and 27% depending on the population, type of ICU, and diagnostic criteria applied [2,3]. VAP prolongs the hospitalization stay and need for mechanical ventilation, which considerably increases costs [4,5]. VAP-associated mortality rates vary between 20% and 70%, and are even higher when caused by multiresistant pathogens or when inadequate antibiotic therapy is used [6,7].

VAP pathogenesis may include aspiration of oropharyngeal mucous, tracheal intubation, inadequate handling of airways, contaminated inhalation, hematological dissemination of a distant infectious area, and exogenous contamination of the pleural space [3,7]. Patients’ characteristics and the treatment they receive may also favor the development of VAP [6].

Bacterial resistance is an important factor associated with increasing mortality rates in patients with VAP. The main VAP-multiresistant bacteria are Gram-negative organisms, such as *Pseudomonas aeruginosa*, *Acinetobacter baumannii, Klebsiella pneumoniae, Enterobacter* spp*.*, and *Escherichia coli.* However, the incidence of methicillin-resistant *Staphylococcus aureus* (MRSA) has gradually increased [4,6,8]. Increasing detection of these bacteria in association with VAP has been attributed to the widespread use of antimicrobials, prolonged use of IMV in the ICU, and the presence of comorbidity [9-11].

Determination of the VAP-associated epidemiology in each medical institution is important for developing effective prophylactic and therapeutic strategies aiming to decrease the incidence of VAP-associated mortality rates, and also to optimize the use of antimicrobial agents [12-14]. In this study, we identified the epidemiological aspects associated with VAP in the ICU of a public hospital in Amapá, Brazil, during June 2009 to May 2010.

## Methods

### Study design and settings

This was an analytical descriptive prospective cohort study performed in an ICU with 11 beds in a general public hospital in Macapá, Amapá, northern Brazil. The study was performed between June 2009 and May 2010 with patients undergoing mechanical ventilation. This is a reference hospital for adult surgery and medical clinics. This hospital has 50 surgical beds, 98 beds for clinics, and 11 isolated beds, as well as the 11 beds in the ICU.

### Inclusion criteria

All patients hospitalized in the ICU who developed pneumonia after 48 hours of invasive mechanical ventilation were included in the study. Patients were followed until discharge from the ICU or until death. Only the first episode of VAP was considered for each patient.

### Ethics

Informed consent and approval by the Ethics Committee were waived because of the observational nature of the study (Ethics Committee in Research of the Medical School Seama in Macapá, Amapá, Brazil, number 030/09).

### Data collection

The following data were collected from VAP patients: age, sex, cause for hospitalization in the ICU (clinical or surgical), presence of comorbidities, date of ICU hospitalization, and date IMV was initiated. VAP was classified as late-onset (>4 days) or early-onset (≤4 days) [2]. Exposure to risk factors was also evaluated. These risk factors were as follows: consciousness level according to the Glasgow scale; transfusion of more than four units of hemoderivates in the last 10 days; antibiotic therapy during the 15 days prior VAP; tracheostomy and hemodialysis for at least 48 hours; invasive procedures (probes, catheters or drains); hospitalization; IMV; and pneumonia in the last 30 days. Empirical antibiotic therapy initiated after VAP treatment was also evaluated for its adequacy according to culture and susceptibility test results.

Bacterial cultures were classified as mono or polymicrobial (>1 microorganism isolated). The time under IMV and the duration of ICU hospitalization were also considered.

### Microbiological diagnosis and susceptibility test to antimicrobial agents

At the time of diagnosis, tracheal secretion was collected from patients through an aspiration sterile procedure using a probe connected to a Transbac C® system (Probac, São Paulo, Brazil), according to the manufacturer’s recommendations. Collection was performed before the antimicrobial agent in use was replaced by a different agent. Samples were sent to the microbiology lab for quantitative culture in blood agar and in MacConkey agar. After overnight incubation under appropriate conditions, the growth was quantified. Cultures were considered positive when growth of at least 10^5^ CFU/mL was observed [15]. All cultures were submitted to bacterial identification and a susceptibility test to antimicrobial agents using the automated Vitek 2 system (bioMérieux).

### Definitions

We applied the diagnostic criteria described by the CDC (1988) to define VAP, which is based on the presence of more than one clinical sign associated with a radiological sign. The clinical criteria included a body temperature of >38°C or <36°C with no other known cause, and/or <4.000 or >12.000/mm^3^ leucocytes. Other criteria also included at least one of the following signs: purulent tracheal secretion or a change in characteristics of an existing secretion; suggestive auscultation and culture of tracheal secretion positive at a threshold of 10^5^ CFU/mL. Radiological criteria included new or progressive pulmonary infiltrates, or consolidation or cavitation in chest radiography [1].

Antibiotic therapy was considered adequate when initiated no later than 48 hours after VAP diagnosis and when it included at least one antimicrobial agent to which the etiological agent was described as susceptible in the antibiogram result [10]. Previous antibiotic therapy was characterized by the use of antimicrobial agents for more than 24 hours during a 15-day period before the VAP episode [11].

Classification of multiresistant bacteria included MRSA, extended-spectrum β-lactamase-producing enterobacteria, *P. aeruginosa*, and other nonfermenting organisms (*A. baumannii* and *Stenotrophomonas maltophilia*) resistant to three or more of the following classes of antibiotics: antipseudomonal cephalosporins or penicillins, carbapenems, fluoroquinolones, and aminoglycosides [10].

### Statistical analysis

Data were analyzed using the software Bioestat version 5.0 [16]. The Shapiro-Wilk test was used to verify the normality of the numeric variables. The G-test of independence was used to evaluate microbial frequency and bacterial resistance with death. Pearson’s correlation was used for numerical variables with normal distribution. Abnormally distributed variables were analyzed by Spearman’s correlation. Significance was accepted when *P* < 0.05.

## Results

Of 126 patients submitted to IMV for at least 48 hours, 33 (26.2%) developed VAP while in the ICU during June 2009 to May 2010. Death occurred in 26 (78.8%) cases (data not shown). Table 1 shows the clinical and epidemiological characteristics of these 33 patients who developed VAP. For all variables tested, we found that only the presence of comorbidity showed a significant association (*P* = 0.029) with death (Table 1).

**Table 1:** Characteristics of 33 VAP patients after ICU admission in a public hospital of northen Brazil

**Characteristics**	**Deceased (n = 26)**	**Survivors (n = 7)**	**p-value**
Categorical variables, n (%)			
Gender, male	20 (76.9)	4 (57.1)	0.3122
Cause of ICU admission			
Clinical	12 (46.2)	3 (42.9)	0.7859
Surgical	14 (53.8)	4 (57.1)	
Comorbidities	16 (61.5)	1 (14.3)	0.0291*
Glasgow < 9	18 (69.2)	4 (57.1)	0.8808
Late-onset VAP	17 (65.4)	5 (71.4)	0.8808
Previous pneumonia	9 (34.6)	1 (14.3)	0.5552
Invasive procedures			
Nasogastric probe	25 (96.1)	7 (100)	0.5177
Vesical probe	26 (100)	7 (100)	1.000
Central vascular catheter	19 (73.1)	4 (57.1)	0.7285
Hemodialysis	6 (23.1)	1 (14.3)	0.9874
Tracheostomy	10 (38.5)	5 (71.4)	0.2590
Prior IMV	10 (38.5)	1 (14.3)	0.4385
Appropriate antibiotic therapy	6 (23.1)	4 (57.1)	0.2147
Polymicrobial etiology	9 (34.6)	1 (14.3)	0.5552
Transfusion of hemoderivatives ≥ 4	5 (19.2)	2 (28.6)	0.9874
Previous use of antibiotics	25 (96.2)	7 (100)	0.5177
Continuous variable, median (interquartile range)			
Age (years)	59 (48.5 – 68.8)	33 (28 – 59)	0.2466
Hospital stay (days)	30 (12.5 – 47.3)	48 (43 – 55.5)	0.0613
Duration of IMV (days)	22 (11.3 – 44)	28 (24.5 – 30.5)	0.7916

IMV: invasive mechanical ventilation; VAP: ventilator-associated pneumonia; CVC: central venous catheter; ICU: intensive care unit. *p < 0.05.

Table 2 shows the distribution of microorganisms isolated after quantitative cultures, and their association with drug multiresistance and patient death. For the 32 samples for which lab results were obtained (one sample was missed), 22 (68.8%) showed one etiological agent and 10 had polymicrobial culture (31.2%), in which up to three different microorganisms were isolated. Thirty-nine species of bacteria were detected, including *P. aeruginosa* (11/39, 34.4%), *Acinetobacter* spp. (11/39, 34.4%), and members of the Enterobacteriaceae family (*Klebsiella*, *Enterobacter*, and *E. coli:* 8/32, 25%) as the most frequent. The difference in the species frequency in VAP was significant (*P* = 0.003). Twenty-three of the 39 (59%) bacterial species were multiresistant. At least one type of multiresistant bacteria was found in 20 (62.5 %) patients. A high frequency of multiresistance was found in *Acinetobacter* spp. (81.8%), *S. aureus* (80%), and *P. aeruginosa* (72.7%). A significant association was found between the frequency of these microorganisms and death (*P* = 0.0001), and also between the presence of multiresistance and death (Table 2, *P* = 0.001). Comparison of bacterial multiresistance and death in VAP patients with death in VAP patients infected with susceptible bacteria did not show any association (*P* = 0.211). No difference was observed for the ratio of multiresistant bacteria between early-onset VAP and late-onset VAP (data not shown).

**Table 2:** Frequency of multiresistant bacteria from 32 VAP patients and their relation to death

**Microorganism**	^**a **^**Frequency n (%)**	^**b **^**Multiresistance n (%)**	**Death n (%)**
Gram-positive			
*Staphylococcus aureus*	5 (15.6)	4 (80.0)	5 (100)
Gram-negative			
*Pseudomonas aeruginosa*	11 (34.4)	8 (72.7)	8 (72.7)
*Acinetobacter* spp.	11 (34.4)	9 (81.8)	9 (81.8)
*Klebsiella* spp.	5 (15.6)	2 (20.0)	4 (80.0)
*Stenotrophomonas maltophilia*	2 (6.2)	-	1 (50.0)
*Sphingomonas paucimobilis*	2 (6.2)	-	1 (50.0)
*Escherichia coli*	1 (3.1)	-	1 (100)
*Enterobacter* spp.	2 (6.2)	-	2 (100)
*P* value (G test) ^c^	0.003	0.0083	
Total	39	23	31

^a^ Spearman correlation between death and frequency of microorganisms (ρ = 0.905, *P* = 0.0001).

^b^ Spearman correlation between death and presence of multiresistance (ρ = 0.947, *P* = 0.00144).

^c^ G Test of independence is evaluating the proportion of different microorganisms among them.

Three species of fungus were found in four tracheal samples. Of these, two presented with *Candida albicans* in association with MRSA, and one patient presented with *Candida parapsilosis* in association with a multiresistant *Klebsiella* strain. One strain of *Cryptococcus laurentii* was isolated as the sole pathogen in one patient who died (data not shown).

The resistance profiles of major Gram-negative bacteria to selected antimicrobial agents tested are shown in Table 3. The resistance ratio varied among antibiotics (χ^2^: 58.4, *P* < 0.0001), piperacillin (63.3%), cefepime (53.3%), aztreonam (50%), ceftazidime (50%), and ciprofloxacin (50.0%) were associated with most of the resistance. Those Gram-negative bacteria were less resistant against amikacin (20.0%), tobramycin (33.3%), and piperacillin/tazobactam (33.3%). *P. aeruginosa* and *Acinetobacter* spp. showed high levels of resistance against carbapenems, cephalosporins, and fluoroquinolones. The resistance ratio of *S. aureus* also varied among antimicrobial agents (G = 66.19, *P* < 0.0001). All isolates showed resistance to penicillin, erythromycin, clindamycin, and tetracycline, and were susceptible to vancomycin, quinupristin/dalfopristin, and linezolid (data not shown). The minimum inhibitory concentration of all *S. aureus* strains for vancomycin and linezolid were 1 μg/mL and 0.5 μg/mL, respectively. Four strains (80%) were also classified as MRSA.

**Table 3:** Resistance profiles of major Gram-negative bacteria from 27 VAP patients in a public hospital of northern Brazil

**Microorganism**	**N**	**Antibiotics**^**a**^**/No. of resistant strains (%)**										
		**PIP**	**FEP**	**ATM**	**CAZ**	**CIP**	**MEM**	**GEN**	**IPM**	**TZP**	**TOB**	**AMK**
*P. aeruginosa*	11	5 (45.4)	6 (54.5)	4 (36.4)	6 (54.5)	5 (45.4)	5 (45.4)	4 (36.4)	5 (45.4)	4 (36.4)	4 (36.4)	2 (18.2)
*Acinetobacter* spp*.*	11	7 (63.6)	7 (63.6)	8 (72.7)	7 (63.6)	7 (63.6)	7 (63.6)	5 (45.4)	6 (54.5)	5 (45.4)	2 (18.2)	3 (27.3)
Enterobacteriaceae^b^	8	7 (87.5)	3 (37.5)	3 (37.5)	2 (25.0)	3 (37.5)	1 (12,5)	4 (50.0)	1 (12,5)	1 (12,5)	4 (50.0)	1 (12,5)
Total	30	19 (63.3)	16 (53.3)	15 (50.0)	15 (50.0)	15 (50.0)	13 (43.3)	13 (43.3)	12 (40.0)	10 (33.3)	10 (33.3)	6 (20.0)

^a^ PIP: Piperacillin; ATM: Aztreonam; FEP: Cefepime; CAZ: Ceftazidime; CIP: Ciprofloxacin; MEM: Meropenem; GEN: Gentamicin; IPM: Imipenem; TZP: Piperacillin/Tazobactam; TOB: Tobramycin; AMK: Amikacin. ^b^*Klebsiella* (5), *Enterobacter* (2), and *Escherichia* (1).

Previous antibiotic therapy was used in 97% of the patients with VAP. The antibiotics most commonly used were cefepime (44.4%), ceftriaxone (25.9%), ciprofloxacin (25.9%), and imipenem (22.2%). A high, positive, and significant correlation was observed (r = 0.8622, *P* = 0.0272) between previous antibiotic therapy and bacterial resistance displayed by Gram-negative bacteria (Table 4).

**Table 4:** Correlation between previous antibiotic therapy and bacterial resistance displayed by Gram-negative bacteria

**Antibiotic**	**Patients with previous antibiotic therapy n (%)**	**Bacteria resistance n (%)**	r ( *P *)*
Cefepime	12 (44.4)	7 (58.3)	0.8622 (0.0272)
Ceftriaxone	7 (25.9)	7 (100.0)	
Ciprofloxacin	7 (25.9)	5 (71.4)	
Imipenem	6 (22.2)	2 (33.3)	
Amikacin	2 (7.4)	-	
Ceftazidime	1 (3.7)	1 (100.0)	

* Spearman correlation.

## Discussion

The frequency of VAP mainly varies according to the diagnostic criteria applied and to the type of ICU and population. According to the criteria applied in this study, the frequency of VAP was of 26.2%. Other authors have found incidences between 9% and 27%, and this incidence increases according to the duration of mechanical ventilation [17].

The mortality rate found in patients undergoing VAP in a few studies performed in Brazil varies between 32.1% and 70.9% [18-21], which agrees with a review by Chastre and Fagon who reported rates from 24% to 76% [6]. The present study found a higher rate of mortality (78.8%) than previous studies, and that comorbidity was the only variable significantly associated with mortality in VAP patients. The presence of some underlying conditions or diseases, including cardiac surgery, acute lung injury, and immunocompromising conditions, significantly affect prognosis in VAP patients, and consequently, the mortality rates are high [6].

Distinct microorganisms are described as agents of VAP, and their frequency may differ according to many factors, including the population of patients in the ICU, duration of hospital stay, and methods of diagnosis. However, in many studies, Gram-negative bacteria were involved in more than 60% of VAP cases, with principally *P. aeruginosa*, *Acinetobacter* spp., and Enterobacteriaceae. Among Gram-positive bacteria, *S. aureus* is the predominant agent in VAP cases [4,10,11,22]. Results from Latin American studies are similar to ones obtained in this study [18,20,21,23,24]. *S. maltophilia* has also been frequently described in VAP cases [11,13]. Similar to these previous studies, in our study, the most frequent microorganisms were *Acinetobacter* spp., *P. aeruginosa*, Enterobacteriaceae, and *S. aureus*. Their frequencies were also significantly associated with death. Three isolates of *Candida* were detected, but they did not appear to be involved as etiologic agents because they were isolated in association with multiresistant bacteria, and because yeasts have been detected from respiratory tract specimens from patients without disease [25,26].

Microbiological investigation of VAP is of great importance for developing appropriate antimicrobial therapy and for standardizing empirical therapies to be used in the future. This is because the local susceptibility profile of bacteria commonly associated with the disease would already be known. In this context, the culture of tracheal aspirate has similar importance for diagnosis compared with invasive techniques of bronchoalveolar wash and a protected specimen brush, and it is also a simpler and less expensive technique [27]. However, quantitative cultures of tracheal aspirates have low specificity (48%–78%) and sensitivity (26%–65%) [8]. In our study, the mortality rate was high, even though etiological agents were identified in 97% of the cases with quantitative culture of the tracheal aspirates, confirming that others factors are important in the prognosis of VAP patients, as previously described [6].

In our study we found a frequency of 59% of multiresistant bacteria associated with VAP. In another study in which the criteria applied for classifying multiresistant bacteria were similar to ours, the frequency of multiresistant bacteria was 27% [10]. Among the most frequent multiresistant bacteria found in VAP are *P. aeruginosa, Acinetobacter* spp*.,* and *S. aureus*[10,12,19]. This finding was confirmed in our study.

Because we analyzed the isolation frequency of those bacterial species that were multiresistant, a significant correlation with death was observed. However, no association was evident when we compared death in patients displaying multiresistant isolates with death in patients with VAP caused by non-multiresistant bacteria, which is the ideal comparison. The lack of association between antibiotic resistance and mortality has been previously reported in VAP patients whose agents were *P. aeruginosa* and MRSA [28,29]. However, bacterial resistance was responsible for a high mortality rate in patients undergoing VAP in many reports [9,30-32]. Such lack of association observed in the current study is probably due to the small sample size, which is a limitation of this study.

For bacterial resistance, it is difficult to determine the most appropriate antimicrobial therapy for VAP, and patients also stay for long periods in hospitals and ICUs, where use of antimicrobial agents is greatly required [24,33]. Additionally, the likely multiresistant bacterial etiology of VAP increases the use of large spectrum empirical antibiotics and of combined therapies [10]. The high levels of antibiotic resistance found among *P. aeruginosa*, *Acinetobacter* spp., and *S. aureus* against major antibiotics usually administered in patients infected with these microorganisms could have led to inadequate antibiotic therapy and a poor outcome in our patients.

In our study, we found that previous antibiotic therapy was used in 97% of patients with VAP. The antibiotics most used were cefepime, vancomycin, and cefalotin. According to the spectrum range of antimicrobial agents for Gram-negative bacteria, carbapenems present with the largest spectrum range of action, followed by cefepime, piperacillin/tazobactam, quinolones, and others [13]. Depuydt *et al.* observed that patients who received antibiotics of at least two different classes showed a higher probability of getting infected by multiresistant bacteria [10]. Previous use of antibiotic therapy also affects the resistance of etiological agents of VAP [11]. Occurrence of multiresistant *P. aeruginosa* and MRSA has been significantly associated with previous exposure to ceftazidime, while *Acinetobacter* spp. are associated with previous exposure to piperacillin/tazobactam [9], which are similar findings to our study.

## Conclusions

In our study, we found that VAP in our ICU presented with high frequency and was mainly caused by multiresistant bacteria. High mortality rates were found to be associated with the frequency of certain bacterial species and with the presence of comorbidities, while the use of previous antimicrobial agents affected the resistance of etiological agents of VAP.

The implementation of rational protocols for the use of empirical antibacterial agents, based on the knowledge of local microbiological patterns, and rapid delivery of culture and results of susceptibility assays are essential strategies that may help in decreasing VAP-related mortality rates by multiresistant bacteria in the ICU.

## Competing interests

The authors declare that they have no competing interests.

## Authors’ contributions

MMR conceived the study, carried out the data collection, organized data, and drafted the manuscript. SGM participated in the design of the study and performed the statistical analysis. BC carried out the data collection, organized data, and drafted the manuscript. PMSF was involved in analysis of microbiological data. CRAVM was involved in analysis of clinical data and reviewed the manuscript. VMN conceived the study, participated in its design and coordination, and helped draft the manuscript. All authors read and approved the final manuscript.

## Pre-publication history

The pre-publication history for this paper can be accessed here:

http://www.biomedcentral.com/1471-2334/13/119/prepub
